# Breast Cancer Dataset, Classification and Detection Using Deep Learning

**DOI:** 10.3390/healthcare10122395

**Published:** 2022-11-29

**Authors:** Muhammad Shahid Iqbal, Waqas Ahmad, Roohallah Alizadehsani, Sadiq Hussain, Rizwan Rehman

**Affiliations:** 1 Department of Computer Science and Information Technology, Women University AJK, Bagh 12500, Pakistan; 2 Higher Education Department Govt, AJK, Mirpur 10250, Pakistan; 3 Institute for Intelligent Systems Research and Innovation (IISRI), Deakin University, Geelong, VIC 3216, Australia; 4 Examination Branch, Dibrugarh University, Dibrugarh 786004, India; 5 Centre for Computer Science and Applications, Dibrugarh University, Dibrugarh 786004, India

**Keywords:** breast cancer diagnosis, malignant growth, deep learning, machine learning, tumor

## Abstract

Incorporating scientific research into clinical practice via clinical informatics, which includes genomics, proteomics, bioinformatics, and biostatistics, improves patients’ treatment. Computational pathology is a growing subspecialty with the potential to integrate whole slide images, multi-omics data, and health informatics. Pathology and laboratory medicine are critical to diagnosing cancer. This work will review existing computational and digital pathology methods for breast cancer diagnosis with a special focus on deep learning. The paper starts by reviewing public datasets related to breast cancer diagnosis. Additionally, existing deep learning methods for breast cancer diagnosis are reviewed. The publicly available code repositories are introduced as well. The paper is closed by highlighting challenges and future works for deep learning-based diagnosis.

## 1. Introduction

Computational pathology (CP) has the potential to improve clinical workflow efficiency and diagnostic quality thanks to information integration and advanced digital communication networks [1]. CP is accompanied by several challenges, such as efficient data fusion, limited processing capabilities, and compliance with ethical practices [2].

Over 2 million women were examined for breast cancer in 2018, among whom approximately 0.6 million died worldwide. Most intrusive breast cancer diseases are chemical receptor-positive [3]. Chemical therapies targeting the trauma center flagging pathway often help patients with chemical receptor-positive tumors [4]. After delicately segmenting a patient’s example onto magnifying instrument slides for staining, a pathologist draws a visual conclusion based on hematoxylin and eosin (H&E) staining, and subatomic marker-explicit stains are used for confirmation and subtyping. Trauma centers are identified using atomic ImmunoHistoChemistry (IHC). However, IHC staining is both time-consuming and expensive [5,6]. Moreover, test quality can vary significantly due to differences in tissue, the skill level of the expert taking the tissue sample, and specialist ability levels [7,8]. Finally, pathologists’ decisions are prone to error [9]. These factors contribute to misdiagnosis. About 20% of current IHC-based trauma center and PR test results are incorrect [9,10], putting patients at risk of receiving subpar treatment. Recent research has shown that emergency room tests can be resolved using morphological stains. However, these studies rely on single-focus tissue microarray datasets (TMAs) [11].

This review examines the application of deep learning (DL) in understanding breast cancer images. We start by pointing out the significance of imaging in nervous system science and its clinical advantages. The review is continued by discussing DL advancements in breast cancer diagnosis. The capabilities of such frameworks, their challenges and possible solutions, and related datasets are investigated. The primary contributions of this paper are:

- Recent articles (from 2018 to 2022) regarding the application of DL in breast cancer diagnosis are reviewed.
- Open datasets related to breast cancer diagnosis are introduced, and their web links are given.
- Publicly available source codes related to existing papers are listed with their web links.
- Current challenges and possible future direction are given regarding the application of DL in breast cancer diagnosis.

The rest of the paper is organized as follows: a brief introduction of digital pathology, breast cancer, and the potential of artificial intelligence (AI) to automate the diagnosis process is given in Section 2. A set of datasets, existing literature, and challenges in breast cancer diagnosis using DL are given in Section 3. Discussion is presented in Section 4 followed by the conclusion in Section 5.

## 2. Digital Pathology and Deep Learning

Pathology is represented by a variety of terms, including “computerized pathology”, “AI”, and “computational pathology”. With the advancement of fluorescent slide scanners, entire glass slides can be virtualized and digitized [12]. The data from the slides can be saved in cloud storage, allowing pathologists to analyze the data with ease and the benefit of assistance from AI-based diagnosis tools [13,14]. To this end, researchers have already developed a variety of AI methods for medical diagnosis [15].

Breast cancer is the most widely recognized malignant growth in women, accounting for nearly half of cancer cases diagnosed in women [16,17]. HR-positive and lymph hub-negative infections also account for nearly half of all cases [18,19,20]. Following widespread clinical approval, multigene tests such as the Oncotype DX 21-gene test, PAM50, and Mamma Print are used to examine patients and guide ACTx in HR-positive and lymph node-negative breast cancer [21,22]. The clinical benefit of the 21-gene test is debatable in patients with HR-positive, lymph hub-negative, and early-stage breast cancer [23,24]. Furthermore, the fragility of RNA extracted from formalin-fixed paraffin-inserted (FFPE) tissue may jeopardize its precision and prevent proper interpretation of recurrence score (RS) results [24]. As a result, a simpler and more effective strategy for determining the risk of repetition based on super-durable tissue is required. Considering that the RS from the 21-gene test is not entirely determined by the expansion qualities bunch score (MKI67, STK15, BIRC5, CCNB1, and MYBL2) and that the mitotic count is linked to the RS7, a careful obsessive evaluation of mitosis and other cell-cell collaborations includes the RS7. Recently, the Lunit Extension has been demonstrated to predict mitosis accurately in every cell in bosom malignant growth [25], as well as recognized cancer cells and other cells in a microenvironment.

Breast carcinoma is the most common malignant growth in women worldwide, and it encompasses a wide range of diseases with varying histological, prognostic, and clinical outcomes [26,27]. Metastatic infections, such as liver and cellular breakdowns in the lungs, affect a majority of patients with malignant bosom growth [28]. A comprehensive genomic analysis of bosom disease patients identified key drivers of hereditary transformations responsible for therapeutic ramifications and outcome prediction [29].

## 3. Automated Breast Cancer Diagnosis

Inspired by the working mechanism of the human brain, artificial neural networks (ANNs) exploit multi-layer complex neuron structures to achieve high representation power [30]. Promising results of ANNs encouraged researchers to develop convolutional neural networks (CNNs) to handle high dimensional data such as images [31,32]. Thanks to automatic feature extraction using convolutional and max pooling layers, CNNs are able to learn challenging tasks [33,34].

### 3.1. Search Strategy

**Figure 1:**
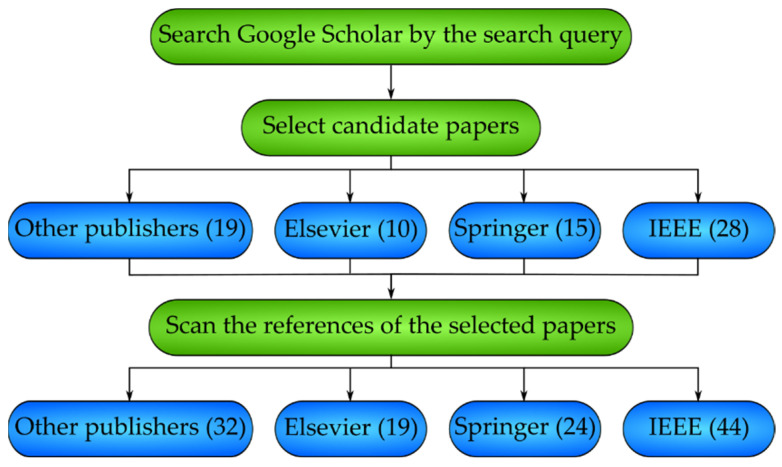
The statistics of the selected papers are categorized according to their publishers.

In this section, the search strategy for gathering existing papers related to breast cancer diagnosis is explained. To conduct our search, an AND/OR combination of multiple keywords was used: (breast cancer diagnosis OR malignant growth OR tumor) AND (deep learning OR machine learning). A total of 514 papers were gathered. Inclusion/exclusion of the gathered papers was performed based on authors’ voting. Papers with at least three votes were considered for inclusion in this survey. The number of selected papers categorized by their publishers were 10, 15, 28, and 19, corresponding to Elsevier, Springer, IEEE, and other publishers. These statistics correspond to the first blue row of Figure 1. We repeated our search among the references of the selected papers. Among the selected papers, 9, 9, 16, and 13 belonged to Elsevier, Springer, IEEE, and other publishers, which have been added to the statistics in the first blue row of Figure 1 to yield the values in the second blue row of the same figure.

### 3.2. Breast Cancer Datasets

There are multiple publicly available datasets for breast cancer diagnosis. To aid cancer detection, some datasets contain viewpoint, malignant growth box, impediment, and other characteristics [35,36]. We undertook extensive research to identify notable breast cancer datasets, which are summarized in Table 1.

### 3.3. DL Application in Breast Cancer Diagnosis

AI has recently demonstrated promising results in terms of precision and accuracy for the automated diagnosis of diseases such as breast cancer [37,38]. Among AI methods, DL stands out for processing high-dimensional data such as medical images [39,40]. An extensive search has been conducted to gather articles related to breast cancer diagnosis. The majority of these articles were gathered from the Nature database, bosom malignant growth. Significant effort has been put into covering recently published articles, especially the ones with publicly available source codes. The remainder of this section is devoted to the overview of the investigated papers.

Wang et al. (the winning team in the CAMELYON16 challenge) created various models using 256 × 256-pixel patches from positive and negative areas of whole slide images of bosom sentinel lymph hubs [41]. Pathologists reported that having a profound learning framework as an assistant decreases the human error rate by 85% [42]. Other studies reported that estrogen receptor status (trauma centers) is a fundamental atomic marker used to diagnose and select treatment options [43,44,45].

**Figure 2:**
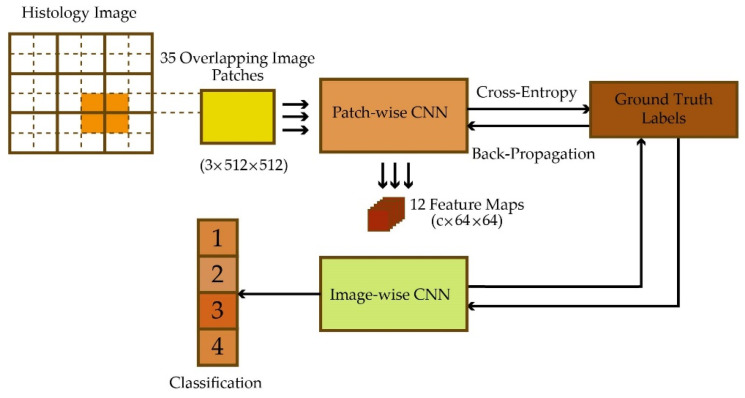
The end-to-end attachment of two networks: 1. Patch-wise CNN, 2. Image-wise CNN.

During clinical administration, pathologists examine biopsied tissue for the designated receptor with immunohistochemistry (IHC) to detect cell surface antigens [46,47]. Due to the importance of tissue analysis, attempts have been made to automate it using DL. For example, two deep neural networks (DNNs) were attached end-to-end for local and global feature extraction from microscopy images [48]. The first network acts as an autoencoder for efficient dimensionality reduction, and the second network takes the job of classification. The steps of this approach are shown in Figure 2.

**Figure 3:**
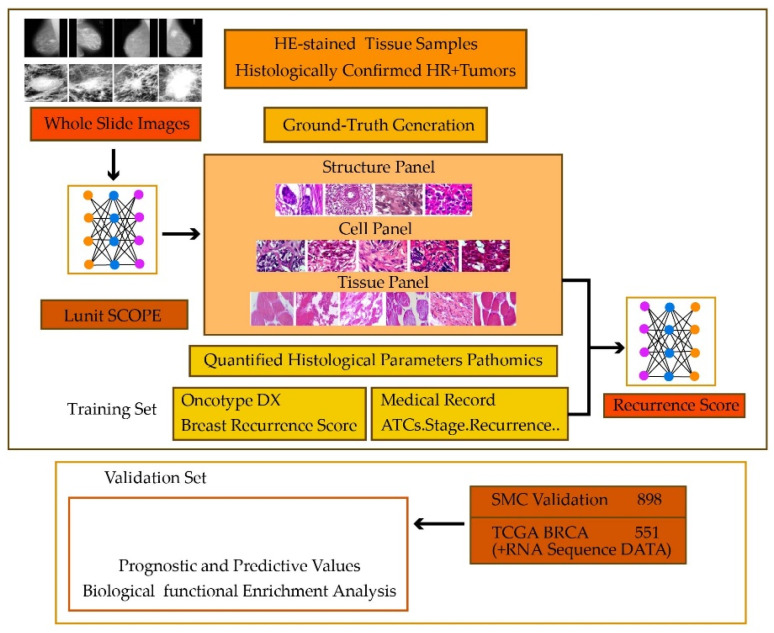
Chemotherapy in hormone receptor-positive breast cancer patients.

Determining the factor with a high impact on cancer patients’ survival is vital for slowing down the cancer progression and increasing the life expectancy of the patients. To this end, Cho et al. [49] investigated the correlation between HE-stained tissue slides and adjuvant chemotherapy benefits for cancer patients. A CNN was trained on 1343 patients to identify histological parameters based on HE-stained whole slide images. The resulting method was called Lunit SCOPE, the steps of which are shown in Figure 3.

**Figure 4:**
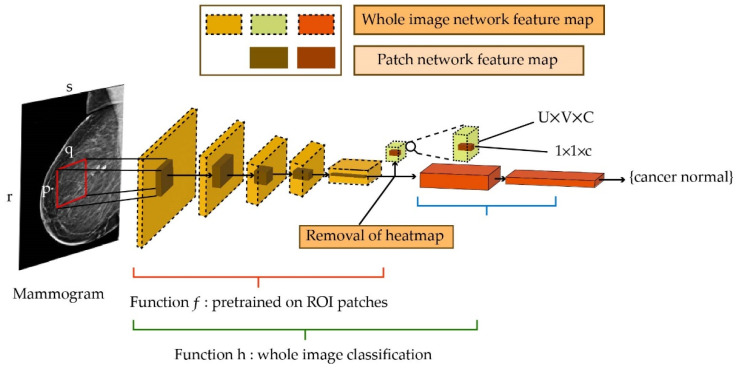
Whole image classification and prediction of cancer or normal.

Another examination approach is a mammogram, which is an X-ray picture of the breast. This approach is even useful for regular examinations of women with no signs of breast cancer. This is particularly important for early diagnosis and taking preventive actions to reduce the potential threat of breast cancer. To this end, Shen et al. [50] utilized DL to diagnose breast cancer based on mammograms. To reduce the cost of preparing a sufficient amount of training data, two sets of training data with different annotations were considered. A limited set of samples with lesion-level annotation was used in the first phase of training. In the second phase, only samples with image-level annotation were used. The cost of image-level annotation is much less than lesion-level annotation, which is appealing. The high-level steps of the aforementioned method are depicted in Figure 4.

**Figure 5:**
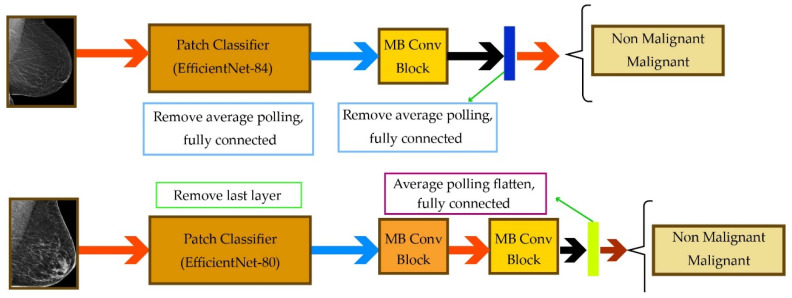
Diagrams of the single-view classifier for the “CV test” (**top**) and “OD test” (**bottom**).

Given that mammography is a reliable approach for breast cancer diagnosis, Petrini et al. [51] have utilized two mammography images (bilateral craniocaudal and mediolateral oblique views) to enhance the diagnosis performance. Their method is based on EfficientNet and has two major components, which are the patch classifier and the whole-image classifier. The patch classifier inspects small sub-images, and the whole classifier uses the patch classifier to scan the whole mammogram. The high-level schematic of this method is depicted in Figure 5. As can be seen, the two mammograms are processed in parallel.

**Figure 6:**
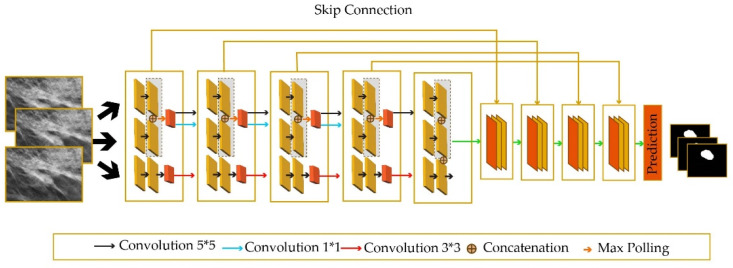
Small Tumor-Aware Network (STAN) to improve the performance of segmenting tumors with different sizes.

In addition to mammography, the detection of small tumors helps with the early diagnosis of breast cancer. To this end, the STAN method [52] has been proposed, which utilizes multiple convolution operations with different kernel sizes to capture breast tumors of various sizes (including small ones). The architecture of STAN is illustrated in Figure 6, in which convolutions with different sizes have been marked with different colors.

Researchers have observed that nuclear protein Ki-67 and tumor-infiltrating lymphocytes (TILs) are important factors for breast cancer diagnosis. Due to the lack of publicly available datasets for Ki-67 stained cell detection, Negahbani et al. [53] gathered such a dataset for public use. Additionally, a DNN named PathoNet was proposed which is a light backbone for cancer diagnosis. To facilitate experimenting with different DL models, a generic pipeline for cancerous cell detection was proposed that is compatible with a variety of DL models.

**Figure 7:**
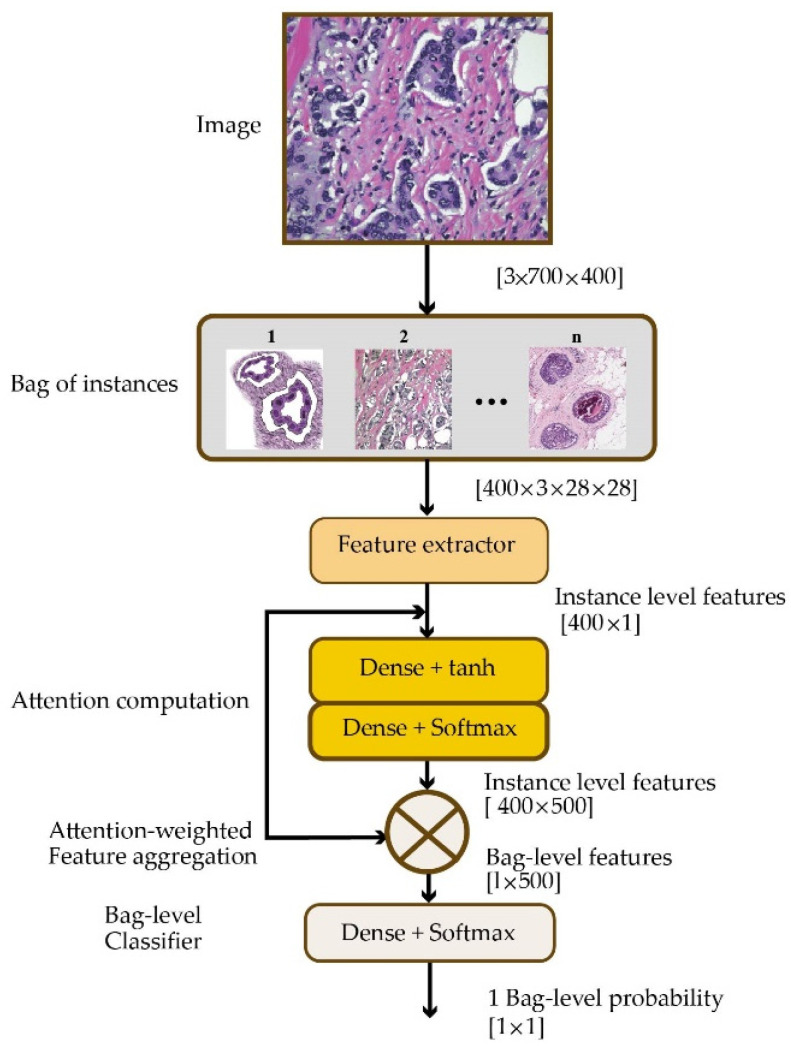
Multi-instance learning architecture for classification of breast cancer histopathology images.

Although achieving state-of-the-art diagnosis performance is important, the ability to interpret the decision-making of DL models should not be overlooked. Being able to reason about the decision-making process is useful to gain better insight into the strengths and weaknesses of DL models. To this end, Patil et al. [54] took a multi-instance learning approach in a weakly supervised manner for the classification of breast cancer histology images. As shown in Figure 7, each input image is partitioned into multiple smaller patches. Feeding these patches to the feature extractor module, attention scores are computed, which are used to compute bag-level features. The classification is performed based on the bag-level features.

**Figure 8:**
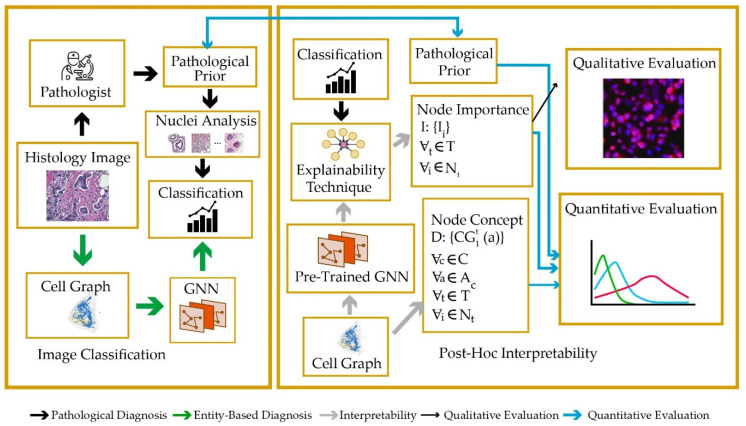
Proposed model based on graph breast cancer identification.

Graph neural networks have also been used to achieve interpretable results from DL models [55]. To this end, a set of quantitative metrics has been proposed to provide pathologists with understandable output. Four graph explainability methods have been used, which are based on graph pruning, gradient-based saliency, and layer-wise relevance propagation. The joint process of classification and explainability data preparation is shown in Figure 8.

**Figure 9:**
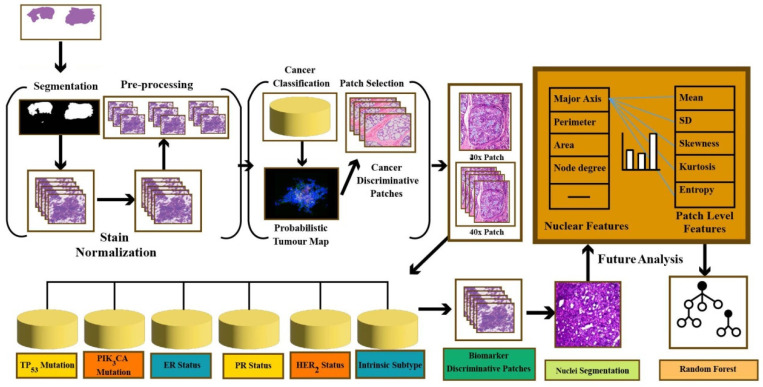
Predict genomic biomarkers—TP53 mutation model.

Despite the considerable potential of DL in the medical domain, medical experts do not fully trust DL. To gain the experts’ trust, the output of DL models must be human-readable (i.e., interpretable). Chauhan et al. [56] have used DL for the prediction of genomic biomarkers such as TP53 mutation, PIK3CA mutation, ER status, etc. The motivation is that classification of genomic biomarkers based on gene expression data is costly and may not be available or sometimes even not feasible. On the other hand, genomic biomarker prediction using DL is an affordable and accessible alternative that is helpful for planning effective treatments. The overall schema of this method is illustrated in Figure 9.

It is also crucial to investigate the effect of using different CNN architectures and hardware processing platforms for breast cancer diagnosis. Such investigation has been undertaken for microscopic images of sentinel lymph tissue [15,57,58]. In particular, Bonnet [59] has conducted careful experiments to evaluate diagnostic performance using different CNN architectures and processing hardware platforms. Moving forward, Bonnet has investigated the effect of using transfer learning, hyperparameter tuning, and data augmentation on the diagnostic performance of DL models.

**Figure 10:**
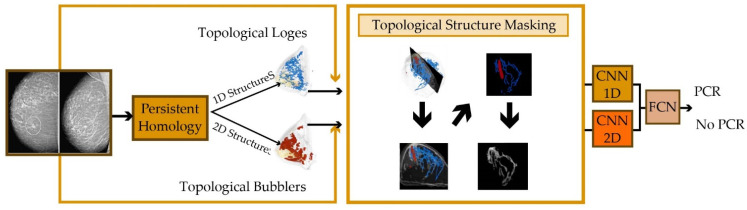
Proposed TopoTxR Method.

Considering that cancer is a chronic disease, monitoring the patient’s status during treatment is critical. Wang et al. [60] have proposed a TopoTxR pipeline for predicting the response to breast cancer treatment. To this end, 1D and 2D topological structures were extracted from breast MRI. Based on these 1/2D structures, new images were created in which voxels corresponding to the extracted structures were set to values in the breast MRI, and the rest were set to zero. The created images were fed to a simple CNN for pathological complete response prediction. The high-level steps of the TopoTxR method are depicted in Figure 10. To facilitate the comparison of existing methods, some of them are summarized in Table 2. Moreover, the set of articles that have accompanying public source codes are gathered in Table 3.

### 3.4. DL Challenges

Despite achieving remarkable results, DL also has its drawbacks [69,70]. To reach acceptable performance, DL methods need a tremendous amount of training data which is hard to come by in the medical domain [71,72]. Preparing training data requires manual labeling which must be carried out by pathologists. This process is costly and requires a considerable amount of time. Moreover, accessing a sufficient number of pathologists may not always be possible [7,73]. Another critical limitation of DL methods is their deterministic nature [74,75]. A well-trained DL model performs well on samples similar to the ones observed during training but fails miserably upon encountering out-of-distribution (OOD) samples. Providing the wrong diagnosis is not acceptable in safety-critical applications such as medical diagnosis. Therefore, it is crucial to develop uncertainty-aware DL models which can estimate how confident they are about their predictions [76,77]. Uncertainty-aware DL has already been investigated in multiple studies [78,79,80], but the field is still an active area of research.

Despite the drawbacks mentioned above, DL has excellent potential for handling challenging tasks [81,82]. For example, in the Camelyon Amazing Test 2016, DL-based approaches were evaluated for disease diagnosis in hematoxylin and eosin (H&E)- stained whole slide imaging (WSI) [83]. Promising outcomes were achieved with a cancer location pace of 92.4%, where a pathologist could accomplish 73.2% responsiveness [10]. Through worldwide joint efforts, computational pathology aims to work on symptomatic exactness, better patient treatment, and treatment cost reduction. Developing better breast cancer diagnosis systems using DL is a crucial part of this objective.

In the last 10 to 15 years, many articles in light of DL have been published [84,85]. Despite significant progress in the field of breast cancer diagnosis, there is still room for improvement. Explainable AI [86] is a research topic that strives to shed light on the complex working mechanism of DL models. Considering that medical diagnosis is safety-critical, careful analysis of DL-based diagnosis systems is an important future aspect [87,88]. Such analysis demands a sufficient amount of annotated data, which is still limited. Therefore, preparing more labeled data is also important for future work [89,90].

## 4. Discussion

Early diagnosis and treatment of breast cancer heavily contribute to increasing life expectancy [91]. In developed countries, age-normalized breast cancer mortality fell by 40% between the 1980s and 2020 [92]. Breast cancer mortality has been reduced by 2 to 4 percent per year in nations that have taken effective treatment strategies [93,94]. Assuming that the breast cancer mortality rate is decreased by 2.5 percent per year, it is anticipated that 2.5 million more patients will stay alive from 2020 to 2040 [95,96].

As a worldwide issue, breast cancer took more than 600,000 lives in 2018. Screening mammography is very effective at reducing bosom disease mortality by 20–40%, and it is recommended by health organizations worldwide for early detection of malignant growth locations [97,98]. Information obtained from our provincial disease reconnaissance framework revealed the status of breast cancer growth endurance and mortality rate in northwestern Iran [99]. Generally, Iran has better breast cancer explicit endurance and a lower mortality rate compared to the country’s general breast cancer growth explicit endurance. However, breast cancer endurance is still lower than in developed nations [100,101].

Breast cancer was reported as the third most common malignant growth in the studies carried out in Iran [102]. The US and Western Europe have reported the highest breast cancer rate, while East Asia has reported the lowest [8,103]. Iran is one of the countries with a rising cancer rate and mortality rate. On the other hand, in agricultural nations, these rates are lower [30]. The aging population, variation to the Western way of life, no full-term pregnancy, late age at first pregnancy, lack of bosom healthcare services, hormonal pregnancy control, and being overweight have contributed to these patterns [104,105,106].

Over the last decade, early diagnosis and efficient treatment have increased the age-normalized life expectancy of patients in developed countries. However, patients in some low-income nations in Africa and Asia still suffer lower life expectancy [87,107]. The Harmony study indicates that the 5-year net endurance rate for bosom malignant growth has consistently increased to almost 80% in numerous nations [108,109]. Breast cancer disease explicit endurance paces of 81–86% have been reported for Britain, Belgium, Canada, the US, and Italy, while comparable figures are much lower in Malaysia (68%), India (60%), Mongolia (57%), and South Africa (53%) [108]. These significant differences might be due to the absence of oncology administrations and medicines, similar to the absence of early diagnosis and screening offices [110]. As indicated by a new Iranian review, the one-, three-, and five-year bosom malignant growth explicit endurance rates were 95.6%, 80.8%, and 69.5%, respectively [111]. Nevertheless, when compared with developed nations, endurance to bosom malignant growth is much lower in Iran, which is partly due to improper treatment modalities [112,113,114,115,116]. It has also been reported that growth size, lymph hub contribution, growth grade, financial status, and genetic inheritance are among the primary factors related to bosom disease explicit endurance [117,118,119]. Disease libraries give basic data to local area-wide anticipation endeavors.

Identifying the major risk factors contributing to breast cancer is crucial for diagnosing breast cancer and controlling its progress. Several studies have been devoted to risk factor identification. For example, Zhang et al. [120] have identified 17 immune genes that were considered prognostic biomarkers for breast cancer. Using these genes and AI, a survival prediction system for breast cancer patients was proposed. Predicting cancer risk as accurately as possible is highly desirable. To this end, Behravan et al. [121] utilized XGBoost [122] to develop an approach for determining the combination of interacting genetic variants and demographic risk factors leading to maximum accuracy in breast cancer risk prediction. Liu et al. [123] have also utilized the XGBoost method to identify risk factors contributing to breast cancer in menopausal women. Given the importance of risk factors contributing to breast cancer, Sharma et al. [124] have devoted a full survey on risk factors and assessment models for breast cancer and pointed out that patients at high risk must receive more frequent examinations.

Automated diagnostic tools not only increase the efficiency of the examination process but also reduce the workload of radiologists. To this end, a commercial AI diagnostic tool was used for breast cancer detection. Based on the AI tool output, the mammograms of patients were triaged in order to reduce the number of patients that undergo radiology [125]. It is possible to prepare models utilizing H&E stains as information and IHC explanations as info marks. This is suitable for multi-instance learning (MIL) [126,127]. Recently, MIL has been utilized to predict ML-driven histopathology [128].

ML approaches for medical diagnosis need to be interpretable, i.e., they must be able to specify the regions of interest in the image. Interpretability is fundamental to gaining medical experts’ trust in using automated diagnosis systems based on ML [129,130]. The field of interpretable AI is itself a major research area that is crucial to gaining a better understanding of black box ML models such as DNNs. Based on the nature of the ML model, available data, and interpretation strategy, interpretable AI methods have been categorized [131]. In future work, it is imperative to determine interpretable AI methods best suited for the medical diagnosis domain. The progress toward incorporating interpretability in AI models for medical applications has already started. For example, Karatza et al. [132] have proposed an ensemble of neural networks for breast cancer diagnosis and evaluated its interpretability using individual conditional expectation (ICE) [133] plots. Some other metrics to evaluate the interpretability of AI models are the global surrogate (GS) [134,135] method and the Shapley values (a method borrowed from game theory) [136,137].

## 5. Conclusions

In this review, we looked at the most recent research on breast cancer diagnosis using DL in image modalities. Various well-known DL methods such as CNN, RNN, GoogLeNet, ResNet, and ANN have been used in the literature for breast cancer diagnosis. In addition to reviewing existing DL-based diagnosis methods, the publicly available datasets and source code repositories were introduced as well. Inspection of the existing approaches reveals the significant progress toward automated diagnosis using DL. However, the reliability of these automated systems is yet to be improved before full deployment in real-world applications.

Over the years, the field of DL has made significant progress to the point that model representation power is rarely the limiting factor. However, without having a sufficient number of training samples, these powerful models will be of no use. Dealing with limited training data is an ongoing research field and can be tackled using different approaches. The most obvious way of addressing data shortage is gathering high-quality datasets that are publicly available. However, data collation is not always possible. Image composition is an alternative promising approach that can be used to create new samples by merging two images [138]. In this technique, several background and foreground images are combined in different ways to generate new training samples. Transfer learning is another strategy to deal with data scarcity. It is highly desirable to make transfer learning domain-aware [139]. Oftentimes, existing pre-trained models have been trained on general-purpose datasets such as ImageNet, which bears little resemblance to medical images. To address this issue, it is better to pre-train models on datasets that share common features with our target dataset.

While DL models are general-purpose learners, relying solely on image data is a short-sighted strategy. Investigating the possibility of performance improvement via fusing multiple sources of data [140] is worth investigating. A different but related approach is utilizing an ensemble of DL models for more robust decision making. The challenge is reducing the complexity of ensemble DL models in order to achieve better performance with manageable computational complexity. Knowledge distillation approaches [141,142] may be useful in making ensemble methods computationally efficient without losing much performance.

## Figures and Tables

**Table 1:** Breast Cancer datasets and their links.

Dataset Name	Link
Cancer Waiting Times	https://data.world/datasets/breast-cancer (access: 11 November 2022)
NKI Breast Cancer Data	https://data.world/datasets/breast-cancer (access: 11 November 2022)
Ispy1_Trial	https://data.world/datasets/breast-cancer (access: 11 November 2022)
Mammographic Masses	https://data.world/datasets/breast-cancer (access: 11 November 2022)
Uta4 Datasets	https://data.world/datasets/breast-cancer (access: 11 November 2022)
Breast Cancer Wisconsin (Diagnostic) Dataset	https://www.kaggle.com/uciml/breast-cancer-wisconsin-data (access: 11 November 2022)
Breast Cancer	https://data.world/uci/breast-cancer (access: 11 November 2022)
Seer Breast Cancer Data	https://ieee-dataport.org/open-access/seer-breast-cancer-data (access: 11 November 2022)
Breast Cancer Histopathological Database (Breakhis)	https://web.inf.ufpr.br/vri/databases/breast-cancer-histopathological-database-breakhis/ (access: 11 November 2022)
Atp5b In Breast Cancer	https://www.frontiersin.org/articles/10.3389/fgene.2021.652474/full#supplementary-material (access: 11 November 2022)
Single-Modality And Multi-Modality	https://data.world/datasets/breast-cancer (access: 11 November 2022)
Atp5b In Breast Cancer	https://www.frontiersin.org/articles/10.3389/fgene.2021.652474/full#supplementary-material (access: 11 November 2022)
Breast Cancer Dataset	https://archive.ics.uci.edu/ml/datasets/breast+cancer (access: 11 November 2022)
Breast Cancer Wisconsin (Original) Dataset	https://archive.ics.uci.edu/ml/datasets/breast+cancer+wisconsin+(original) (access: 11 November 2022)
Sklearn.Datasets	https://scikit-learn.org/stable/modules/generated/sklearn.datasets.load_breast_cancer.html (access: 11 November 2022)
Cbis-Ddsm: Breast Cancer Image Dataset	https://www.kaggle.com/awsaf49/cbis-ddsm-breast-cancer-image-dataset (access: 11 November 2022)
Breakhis (Breast Cancer Histopathological Database)	https://paperswithcode.com/dataset/breakhis (access: 11 November 2022)
Mammography Database	http://marathon.csee.usf.edu/Mammography/Database.html (access: 11 November 2022) http://www.eng.usf.edu/cvprg/Mammography/Database.html (access: 11 November 2022)
Cancer Data	https://portal.gdc.cancer.gov (access: 11 November 2022)
Cbioportal	https://www.cbioportal.org/ (access: 11 November 2022)

**Table 2:** Summary of reviewed articles.

Authors	Dataset	Task	Approach	Disease Type	Accuracy (%)
Nikhil Naik et al. [61]	WSI-level annotations	Classification	ReceptorNet	Estrogen receptor Prediction	92
William Lotter et al. [42]	DDSM, OMI-DB	Classification	ResNet-50	detection in mammography	94.5
Bryan He et al. [62]	Histopathology images	Classification	ST-NET	Prediction of local gene expression	66
SooYoun Cho et al. [49]	HE slides	Classification	Lunit SCOPE, CCN	Mitotic cells in the cancer epithelium	50
Li Shen et al. [50]	INbreast data	Detection	CBIS-DDSM	Screening mammograms	95
Farzin Negahbani et al. [53]	SHIDC-BC-Ki-67	Cell detection and classification	PathoNet	Nuclear protein Ki-67 and Tumor infiltrating lymphocytes (TILs)	95.6
Kamyar Nazeri et al. [48]	Breast Cancer Histology (BACH)	Classification	ICIAR, CNN	Tissue, classification microscopy images	95
Abhijeet Patil et al. [54]	BreakHIS, BACH	Classification	A-MIL	HE slides predicts	84.43
Bryar Shareef et al. [52]	BUSIS, Dataset B	Segmentation	STAN	Improve the performance of segmenting tumors	90.2
Guillaume Jaume et al. [55]	BRACS	Detection	Class separability computation	Conventional pixel-wise analysis	62.5
Eric Bonnet [59]	PCam (Patch CAMELYON)	Classification	Shelf deep learning framework	Breast cancer metastasis tissue	89
Fan Wang [60]	e I-SPY1	Classification	TopoTxR	Tissue structures	85.1
Zakaria Senousy [63]	Histology image	Classification	MCUa	Breast histology image classification	98.11
Ruchi Chauhan et al. [56]	TCGA dataset	Classification	Genetic-histologic Relationships	Genomic biomarkers	90.9
Daniel G.P. Petrini et al. [51]	CBIS-DDSM	Classification	EfficientNet-Based Convolutional	Two-View Mammography	87.57
Paul Gamble et al. [64]	FFPE, TCGA and H&E slides	Classification	Two-stage deep learning system (DLS),	Predict ER/PR/HER2, tissue regions (patches)	93.9
Karthik et al. [65]	BreakHis	Classification	Ensemble of DL models	Breast cancer	99.55
Hao et al. [66]	BreaKHis	Classification	Features based on DenseNet201 fused with gray-level co-occurrence matrix, support vector machine	Breast cancer	96.75
VR Allugunti [67]	A Kaggle dataset	Classification	Random forest	Breast cancer	90.55
Wang et al. [68]	Kaggle Histopathologic cancer detection	Classification	Hybrid DL model	Breast cancer	86.21

**Table 3:** Articles that have open-source codes.

Article	Source Codes
[61]	https://github.com/DeepHealthAI/nature_medicine_2020 (access: 11 November 2022)
[42]	https://github.com/ysbecca/py-wsi (access: 11 November 2022) https://github.com/AMLab-Amsterdam/AttentionDeepMIL (access: 11 November 2022) https://github.com/uoguelph-mlrg/Cutout (access: 11 November 2022)
[49]	https://github.com/huiqu18/GeneMutationFromHE (access: 11 November 2022)
[50]	https://github.com/lishen/end2end-all-conv (access: 11 November 2022)
[53]	https://github.com/SHIDCenter/PathoNet (access: 11 November 2022)
[48]	https://github.com/ImagingLab/ICIAR2018 (access: 11 November 2022)
[54]	https://github.com/Dipeshtamboli/Image-Classification-and-Localization-using-Multiple-Instance-Learning (access: 11 November 2022)
[52]	https://github.com/sudohainguyen/STAN-small-tumor-aware-network (access: 11 November 2022)
[55]	https://github.com/histocartography/patho-quant-explainer (access: 11 November 2022)
[59]	https://github.com/erbon7/pcam_analysis (access: 11 November 2022)
[60]	https://github.com/TopoXLab/TopoTxR (access: 11 November 2022)
[63]	https://github.com/zakariasenousy/mcua-model (access: 11 November 2022)
[56]	https://github.com/theRuchiChauhan/biomarker-prediction-breast-cancer (access: 11 November 2022)
[51]	https://github.com/dpetrini/two-views-classifier (access: 11 November 2022)
[64]	https://github.com/tensorflow/tensorflow/tree/r1.14 (access: 11 November 2022)

## Data Availability

Not applicable.
